# Value stream mapping to characterize value and waste associated with accessing HIV care in South Africa

**DOI:** 10.1371/journal.pone.0201032

**Published:** 2018-07-24

**Authors:** Christopher J. Hoffmann, Minja Milovanovic, Anthony Kinghorn, Hae-Young Kim, Katlego Motlhaoleng, Neil A. Martinson, Ebrahim Variava

**Affiliations:** 1 Johns Hopkins University School of Medicine, Baltimore, United States of America; 2 Johns Hopkins University Bloomberg School of Public Health, Baltimore, United States of America; 3 Perinatal HIV Research Unit, Faculty of Health Science, University of the Witwatersrand, Johannesburg, South Africa; 4 Department of Internal Medicine, Klerksdorp Tshepong Hospital Complex and the University of the Witwatersrand, Klerksdorp, South Africa; Julius Centre, University Medical Centre, Utrecht, NETHERLANDS

## Abstract

**Introduction:**

Inefficient clinic-level delivery of HIV services is a barrier to linkage and engagement in care. We used value stream mapping to quantify time spent on each component of a clinic visit while receiving care following a hospital admission in South Africa.

**Methods:**

We described time for each clinic service (“process time”) and time spent waiting for that service (“lead time”). We also determined time and patient costs associated with travel to the clinic and expenditures during the clinic visits for 15 clinic visits in South Africa. Participants were selected consecutively based on timing of scheduled clinic visit from a cohort of HIV-positive patients recently discharged from inpatient hospital care. During the mapping we asked the participants to assess challenges faced at the clinic visit. We subsequently conducted in depth interviews and included themes from the care experience in this analysis.

**Results:**

The 15 clinic visits occurred at five clinics; four primary care and one hospital-based specialty clinic. Nine (64%) of the participants were women, the median age was 44 years (IQR: 32–49), three of the participants had one or more clinic visit in the prior 14 days, all but one participant was on antiretroviral therapy (ART) at the time of the clinic visit (ART was stopped following the hospital visit for that participant). The median time since hospital discharge was 131 days (interquartile range; IQR: 121–183) for the observed visits. The median travel time to and from the clinic to a place of residence was 60 minutes. The median time spent at the clinic was 3.5 hours (IQR: 2.5–5.3) of which 2.9 hours was lead time and 25 minutes was process time (registration, vital signs, clinician assessment, laboratory, and check-out). The median patient cost for transport and food while at the clinic was ZAR43/USD2.8 (median monthly household income in the district was ZAR2450/USD157). Participants highlighted long queues, repeat clinic visits, and multiple queues during the visit (median of 5 queues) as challenges.

**Conclusions:**

Accessing HIV care in South Africa is time consuming, complicated by multiple queues and frequent visits. A more patient-centered approach to care may decrease the burden of receiving care and improve outcomes.

## Introduction

Maintaining engagement in care is critical to the success of global programs for HIV treatment and prevention. Several studies have highlighted the substantial attrition along the care continuum from testing positive to initiating antiretroviral therapy (ART) to achieving an undetectable HIV viral load. Following HIV diagnosis, only 50% or less of patients link to care [1–3] and a substantial proportion subsequently disengage from care [4]. Multiple factors influence HIV care engagement in sub-Saharan Africa including fear, disclosure, stigma, accepting alternative forms of healing, transport challenges, food insecurity, and clinic related factors including cost, time, and inadvertent disclosure associated with clinic attendance [5–8]. Several recent studies have reported on the role of clinic-level factors such as enacted stigma and long queues as creating substantial barriers to engagement in care [9–11]. An additional barrier is the considerable wait time and short contact time with health care providers [12–14].

Clinic-level factors may be an especially large burden for individuals transitioning from diagnosis to chronic care or from hospitalization to community care [10]. Those with recent hospitalization may have additional challenges as they may be regaining mobility and function during their convalescence, making travel to, and long wait times at, clinics especially burdensome. Value stream mapping is a technique developed as part of industrial quality improvement efforts [15] and has been applied to understanding and improving patient services in clinical care, including in Africa [12, 16–18]. Two important barriers to clinic care are long wait times and frequent repeat visits [11, 19]. To quantify how time is spent in obtaining care we used value stream mapping to characterize clinic visits following hospital discharge for people living with HIV in South Africa.

## Materials and methods

This study was conducted according to the principles expressed in the Declaration of Helsinki. Human subject research approval was obtained from the University of the Witwatersrand Human Research Ethics Committee (150613) and the Johns Hopkins Medicine Institutional Review Board (67631). All participants provided written informed consent.

### Setting and study population

Participants for the value stream mapping study were recruited from a cohort of individuals enrolled in a hospital discharge study. This cohort was composed of adults with HIV recruited between April and June 2016 from general medical wards of a single tertiary care hospital in North West Province, South Africa that was the primary hospital for inpatient care in the administrative district. Participants were recruited if they met the following criteria: were ≥18 years of age, spoke one of the study languages (English, Setswana, or isiXhosa), were diagnosed with HIV either prior to admission or during the index hospitalization, were able to complete informed consent or had a legally authorized representative who could perform written informed consent. Among participants in the cohort, those with upcoming scheduled clinic visits, based on the discharge plan, were consecutively contacted no more than one week prior to an anticipated clinic visit date. These visits were generally scheduled to review laboratory results, dispense medications, or review the patient’s clinical symptoms or progress since hospitalization and were generally related to the condition or conditions related to the hospitalization. During contact, participant’s clinic attendance plans were reviewed, if they were planning on going to a clinic during the following week, they were invited to participate in the value stream mapping sub-study. Of note, participants could plan on going to any of the clinics within the administrative district–either the specialty clinic at the tertiary care hospital or one of the 15 primary care clinics. When multiple participants had the same anticipated visit date, the participant with the closest clinic to the study office was contacted first. Only one mapping was done on a given day due to the time and man-power required to observe an entire clinic visit. In addition, mapping did not occur on all days due to other responsibilities of the field workers. Recruitment for value stream mapping ended once 15 observations were completed based on an *a priori* sample size based on operational feasibility.

The primary care clinics were public clinics that provided acute and chronic care services including antiretroviral therapy. Care was primarily delivered by primary care nurses. Post-hospital visits were managed as any other ambulatory care visit without any specific post-hospital triage or protocols for assessment or communication with hospital-based providers.

### Value stream mapping procedures

On the day of the planned clinic visit, field workers met the participants at the clinic and recorded start and stop times for all clinic wait and service times. All patient costs (transportation to and from the clinic, food, and clinic fees) were recorded on a questionnaire administered by the field workers. In addition, the time of departing the place of residence and mode of transport to the clinic were recorded. After each clinic component (e.g. clinician, laboratory) the field worker asked the patient if the primary purpose of that component had been achieved (e.g. obtained vital signs, received clinical assessment, underwent phlebotomy) and if there were any problems or challenges encountered (e.g. no sample tubes for blood draws or a medication stock-out). Participant responses were hand-written by the field worker (sometimes with paraphrasing) and data captured as free-text.

### In depth interviews

In depth interviews were conducted with a sample of 30 participants in the hospital discharge cohort, including participants in the value stream mapping sub-study, to better understand challenges during convalescence. These were conducted on a subsequent day to the value stream mapping, in person, and in a private space. Interviews were audio-recorded and transcribed and translated into English.

### Analysis

We used value stream mapping to describe the pathway of receiving clinic services including wait times and type of encounter [15, 20]. Time was divided into wait time (also known as lead time) and service time (also known as process time). Service times for nurse, nurse clinician, and doctor were aggregated because roles of these health care workers were relatively interchangeable in the study clinics. All time interacting with clinic staff, including time obtaining a file and scheduling follow-up, was classified as service time or time that potentially “added value” to the patient. The use of the term value or adding value in value stream mapping refers to the action directed toward improving health (such as evaluation by a clinician or phlebotomy for laboratory assessments). We considered all waiting time or travel time as lead time as this did not provide a direct service or “value add”. Cost was calculated in terms of the patient-level expenditures to attend the clinic including transport and spending on food. Only two participants encountered clinic fees, these were excluded from calculating median cost; we reported these costs but did not include them in the overall clinic cost because of the small number of participants with clinic fees. We did not assess opportunity costs. Microsoft Excel 2013 (Microsoft Corporation) and Stata 13 (StataCorp LP) were used for analyses.

In addition to the quantitative analysis, we summarized participant’s comments regarding challenges with services that were recorded by the field workers during the value stream mapping. In depth interviews were coded based on the cumulative complexity of care model [21]. We have included quotes specifically on the theme of service delivery from participants who underwent value stream mapping and in depth interviews to include the voice of participants.

## Results

Of the 124 HIV-infected participants in the transition in care study, 14 underwent value stream mapping for 15 clinic visits (one participant had value stream mapping on two consecutive clinic visits) at five different clinics between 9 August and 16 November 2016. Five additional participants were scheduled to meet with a field worker, but the participants did not arrive at the clinic on the scheduled day. The median age was 44 years (interquartile range [IQR]: 32–49), 9 (64%) were women, and median time from the enrollment hospitalization to the observed clinic visit was 131 days (IQR: 121–183), three participants had one or more clinic visit in the prior 14 days, and all but one of the participants were on ART. For one participant this was the first post-hospital visit; the overall median time to the first post-hospital clinic visit was 35 days (IQR: 15, 49). Three participants had an additional hospital admission subsequent to the recruitment index hospital stay and prior to the clinic visit for value stream mapping. Hospital discharge diagnoses were mostly HIV-related, including: pneumonia (2), tuberculous meningitis (1), severe anemia (3), gastroenteritis (2), and seizures (2). Two were diagnosed with HIV during the index hospitalization.

**Fig 1:**
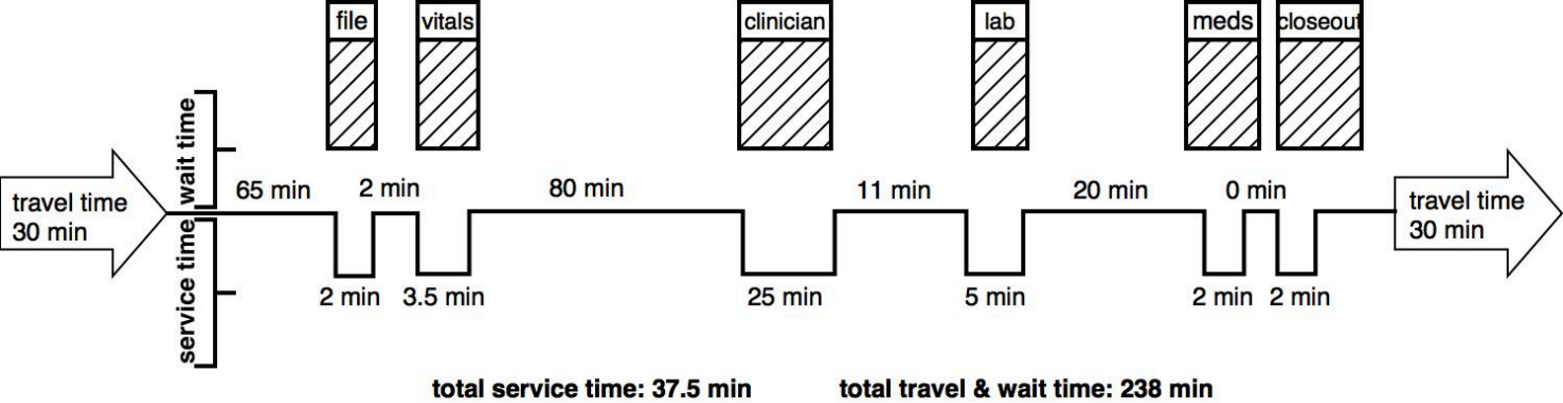
Value stream map of median wait and service times for 15 clinic visits among recently hospitalized people living with HIV in South Africa (not to scale).

The median travel time to and from clinic was 60 minutes with a median cost of ZAR20/USD1.3 (IQR: USD 0–26; Table 1). Of the 15 value stream mappings, ten visits involved public transport, one a private car, and four walking to the clinic. One participant required special wheelchair transport to travel from her residence to clinic. For the 15 clinic visits, one participant had to miss an entire workday from formal employment to go to the clinic, three forwent other income generating activities, eight reported being away from household responsibilities (e.g. child care), and three reported no missed activities for that clinic visit. Due to the long time spent at the clinic, some participants purchased food at a median cost of ZAR23/USD1.5 for a total median cost of transport and food of ZAR43/USD2.8. Two participants had additional costs for opening a file at the clinic, one USD1.3 and the other USD5.1 (this cost was not included in the median cost for all 15 participants). The median total time at the clinic was 3.3 hours (IQR: 2.5–5.3) with a median of 2.9 hours (IQR: 1.3, 5.7) of queue time and 25 minutes (IQR: 10, 49) of service time (registration, vital signs, clinician, laboratory, pharmacy, check-out; Fig 1). Participants waited in a median of 5 different queues (range 1–7) during a clinic visit with the longest queues for registering (65 minutes), seeing a clinician (80 minutes), and receiving medications (20 minutes). The ratio of the patients’ time receiving services to the time spent traveling and waiting was 0.14.

**Table 1:** Value stream mapping results.

Characteristic	Median (IQR) or n
Travel time to clinic, minutes	60 (37, 80)
Time in clinic, hours	3.3 (2.5, 5.3)
Queue time, hours	2.9 (1.3, 5.6)
Number of queues	5 (1, 7)
Service time, minutes	25 (10, 49)
Total time for clinic visit (travel and clinic), hours	4.5 (2.8, 7.3)
Ratio of service time to queue time	0.14 (0.06, 0.35)
Travel mode	
Public transport	10
Walk	4
Private transport	1
Impact on daily earning	
Missed day wage	1
Missed partial day earning	3
Away from non-earning household responsibilities	8
No missed earing or responsibilities	3
Travel cost (USD)	1.3 (0.32, 1.3)
Food cost (USD) for 4 participants who purchased food	1.5 (1.2, 1.7)
Time to next scheduled visit	
0–1 weeks	6
>1–2 weeks	3
3–4 weeks	4
>4 weeks	2

IQR: interquartile range

The median time to the next scheduled clinic visit was 1.5 weeks with 12 participants scheduled to return in less than four weeks and seven in one week or less. Scheduling for the next clinic visit was at the discretion of the provider. Brief intervals to the next visit were generally for repeat evaluation of a frail or particularly ill patient, receiving laboratory results obtained during the observed clinic visit or still pending at the time of that visit, or obtaining medications.

### Challenges clients faced

Five participants specifically complained about the slow movement of queues in the clinic. One participant reported frustration that she was told to return the following day to collect her medications. This was particularly burdensome to her because she had also been to the clinic the previous day. As a result she had three clinic visits on three consecutive days. Three participants had to queue multiple times for the nurse or doctor on the day of the visit because of missing prescription information in their file when they went to collect medications from the pharmacy. One participant reported that queuing was hard for her because she used crutches and struggled to remain standing in the queue. Another participant struggled with walking from queue to queue due to severe dyspnea. Two participants who had been referred to specialists were frustrated because they were turned away by the specialists because inadequate information was included in the referral note.

Themes identified in the in depth interviews reflected similar experiences as observed during the value stream mapping. The two main themes identified were frustration with queueing (long and multiple wait times) and demeaning behavior from clinic staff. For example, one participant described a long queue time (also documented in the value stream mapping) partly due to his name being mispronounced when he was first called so he was unaware that he was next in the queue:

“I still go to the clinic, even though at times they delay with the patient’s files and call people with wrong names. Again, you find yourself arriving at the clinic around 05H45 or 06H45 but you only finish late around 16H00 or 15H00. They sometimes call people with either the wrong names or surname. What happened to me last time really upset me, I was so angry.” (48 year old male)

Another participant required a wheelchair for mobility and described staff rudeness and system inflexibility to accommodate her:

“There is nothing I can do, I would just keep quiet. There is nothing I can do, it’s my date, what can I say? You might tell someone that you have been waiting for long and they shout at you, so I don’t want to be shouted.” (29 year old female)

## Discussion

Our results highlight the complexity and long duration and multiple queues required for typical post-hospital clinic visits for people with HIV in South Africa. The median time at a clinic was 3.3 hours with another one hour for travel; during a quarter of the visits more than 5.3 hours were spent at the clinic. The overall median direct cost (not accounting for opportunity costs) related to clinic visits was ZAR43/USD2.8. This is 1.8% of the median monthly household income in the district where these individuals resided of ZAR2450/USD157 [22]. The typical multiple visits per month following a hospitalization could add substantially to this proportion of household income. A not unexpected finding of this study was the effect of inefficient clinic processes—that appear to be correctable–on waiting times. Training on the requirements of a prescription, ensuring laboratory results are promptly available in the patient record are two that we identified to reduce the need for repeat queueing or clinic visits. Long queues for registration and waiting to see a clinician were also identified. Streamlining the registration, including the process of retrieving the patient file, could reduce an hour from the total time required for care. Identifying ways to reduce the wait time to see the clinician should also be a priority.

This study adds to the literature on this important topic, adding both greater granularity and the post-hospital discharge perspective. A study of prevention of mother to child transmission of HIV (PMTCT) care in clinics in Mozambique reported a mean wait time of 1 ½ to 7 hours and actual consultation time of only 7 to 25 minutes [23]. Another study from all types of visits, also from Mozambique, reported a median wait time for a consult of 43 minutes with only 5.3 minutes of actual service time [13]. Two studies based in HIV clinics in Kenya reported median durations for a clinic visit of 2.1 to 3.2 hours; one of the studies also reported mean time with a doctor or nurse of 15 minutes. [12, 24]. Wait time at an academic referral clinic in Uganda was less, 1.8 hours, and consult time was also less, 10 minutes [25]. A qualitative study using self-report noted a mean time at HIV clinic visits in Tanzania of 6 hours. Other studies have characterized the financial cost of care to the patient due to direct costs and opportunity costs [26]. Our study adds to these findings with granular quantification of time spent for each discrete point of waiting and service at the patient level as well as time to reach the clinic and financial outlays during the clinic visit. Furthermore, we characterized the time from the prior visits and time to next visit for these participants leading to documentation of substantial time and financial burden.

We believe that characterizing lead and service time adds important knowledge to understanding the full burden of clinic visits and how that burden could be reduced while maintaining or improving care. This is important for all clinic patients, but may have special salience for more medically fragile populations such as those with recent acute illness leading to hospital admission. Characterizing the clinic visit from the patient’s perspective is an important step in improving HIV care continuum outcomes as clinic wait time has been identified in both high income and low and middle income countries as associated with care engagement [9, 11, 19, 21]. Furthermore at least one randomized study that markedly reduced the travel time and wait time at public clinics through providing a scheduled home visit, increased care engagement for ART [27]. While long-term health may be a priority for most people living with HIV, patients have to balance competing immediate needs with spending much of the day traveling to and from and queueing at the clinic.

This study has several important limitations. We conducted the study in one district in South Africa, among only 15 clinic visits (due to the labor intensiveness of the data collection). A more accurate description of time and financial costs may have been possible with a larger sample size. However, we believe that the granularity of our approach provides a reasonable and clear representation of the time and financial costs of clinic access at public sector clinics in South Africa. Moreover, we did not seek to perform a complete economic analysis of the cost of care or opportunity costs. The goal of this analysis was to focus on clinic-enacted costs, especially time, borne by the patient and not the wider range of costs surrounding illness in this community. Another limitation is that we generally did not map the first post-hospital clinic visit as the median time to the visits that we observed was 131 days compared to the median time to first clinic visit of 35 days. We do not expect that observations from the subsequent clinic visits would have substantially differed from the initial visit because no specific post-hospital coordination or other services were provided at the first post-hospital visit or at any other visit.

Overcoming current challenges to linkage to HIV care and continued engagement in care in South Africa and globally requires addressing multiple levels of influence. Among the most important may be how and where care is delivered. The fact that only a low proportion of the total time involved in accessing care was related to service delivery highlights current inefficiencies borne by patients. Improving current care delivery is urgently required to increase engagement in care. Systematic follow-up of patients post-hospital through telephonic, in person (home visits), and dedicated clinic services with coordination between inpatient and outpatient care may improve the care experience, follow-up, and patient-centered outcomes [28]. For the many millions of HIV-infected patients in similar settings as those in our study, reducing both the time and financial burden of care would likely improve long term HIV treatment outcomes.

## Supporting information

S1 Process Mapping Data (CSV)
